# Characterization of Microbial Carbon Metabolism in Karst Soils from Citrus Orchards and Analysis of Its Environmental Drivers

**DOI:** 10.3390/microorganisms13020267

**Published:** 2025-01-25

**Authors:** Shixuan Wang, Zhenjiang Jin, Xuesong Li, Hongying Zhu, Fang Fang, Ting Luo, Jia Li

**Affiliations:** 1 College of Environmental Science and Engineering, Guilin University of Technology, Guilin 541006, China; 2120220538@glut.edu.cn (S.W.); 17785145622@163.com (X.L.); zhy990525@163.com (H.Z.); fangfangglut@163.com (F.F.); lt15730527268@163.com (T.L.); 2120220515@glut.edu.cn (J.L.); 2 Guangxi Key Laboratory of Theory and Technology for Environmental Pollution Control, Guilin University of Technology, Guilin 541006, China; 3 Collaborative Innovation Center for Water Pollution Control and Water Safety in Karst Area, Guilin University of Technology, Guilin 541006, China

**Keywords:** karst regions, forest soil, citrus orchards, microorganisms, carbon sources, metabolism

## Abstract

Karst regions (KRs) have created significant karst carbon sinks globally through the carbon cycling process involving “water-carbon dioxide-carbonate rock-biota”. Soil organic carbon (SOC) represents a crucial component of these carbon sinks. Microorganisms play a vital role in the soil carbon cycle, influencing the formation and preservation of SOC. Therefore, investigating the carbon metabolism of soil microorganisms in KRs is essential for clarifying the unique biogeochemical cycling mechanisms within these regions. In this paper, soils from karst regions (KRs), mixed regions (MRs) and non-karst regions (NKRs) were collected from citrus orchards in Mao Village, Karst Experimental Field, Guilin City, Guangxi Zhuang Autonomous Region, China. The ability to use different carbon sources was analyzed by Biolog-Eco microtiter plate technique; the number of microorganisms was detected by the plate colony counting method, and the microbial biomass was determined by the chloroform fumigation method. The results showed that the soil bacterial number (5.69 ± 0.39 × 10^6^ CFU/g), microbial biomass carbon (MBC) (608.24 ± 63.80 mg/kg), microbial quotient (SMQ) (3.45 ± 0.18%), and Shannon’s index (*H*′) (3.28 ± 0.05) of the KR were significantly higher than those of the NKR. The pH showed a significant positive correlation (*p* < 0.05) with the bacterial number and *H*′ (*p* < 0.05); SOC showed a highly significant positive correlation with bacterial number (*p* < 0.01), and a significant positive correlation with MBC, *H*′, and average well change development (AWCD) (*p* < 0.05). Total nitrogen (TN) showed a significant positive correlation with MBC (*p* < 0.05); available potassium (AK) showed a significant positive correlation with bacterial number and MBC (*p* < 0.05). Exchangeable calcium (Ca^2+^) demonstrated significant positive correlations with bacterial number, MBC, and *H*′ (*p* < 0.05). The above results indicate that soil bacterial number, carbon metabolic ability and diversity were highest in the KR. pH, SOC and exchangeable Ca^2+^ were the main influencing factors for the differentiation of soil microbial carbon metabolic diversity between the KR and NKR.

## 1. Introduction

Soil is the second largest carbon sink on Earth and the largest carbon sink in terrestrial ecosystems [1]. Karst landforms cover an extensive area of approximately 22 million km^2^, accounting for about 15% of the earth’s total land surface [2]. The carbon cycling processes in karst regions (KRs) contribute to a global karst carbon sink estimated at around 0.89 Pg C/yr [3], highlighting their significant potential for carbon sequestration. The Guangxi region of China has extensive karst landscapes, in which citrus orchards cover a wide area of 6.31 × 10^3^ km^2^, which constitutes 44.9% of the total orchard area [4] (banana orchards 77.57 km^2^, pear orchards 21.31 km^2^, and vineyards 31.64 km^2^). In recent years, the yield and planting area of citrus have continued to rise annually, making it one of the primary land use types in Guangxi, China. This widespread distribution qualifies it as a representative sample for studying the land use characteristics of karst regions. Research on soil carbon sequestration in citrus orchards within karst areas contributes to our understanding of the fundamental mechanisms of the carbon cycle and enhances the carbon sink function of these regions.

Soil microorganisms serve as crucial drivers of the carbon cycle and significant contributors to carbon balance [1,5]. They engage in multiple processes within the carbon cycle [6]. Soil physicochemical properties such as soil nutrients, pH levels, as well as specific elements (e.g., calcium ions Ca^2+^), are key factors that regulate microbial carbon source metabolic activity and diversity [7,8,9]. Soil organic carbon (SOC) and total nitrogen (TN) are the primary driving forces behind the activities of soil microbial life. These factors significantly enhance soil microbial diversity indices and improve the microbial utilization of carbon sources by influencing microbial community assembly and dynamics. In turn, this affects the metabolic activity and diversity of soil microbial carbon source utilization [7,10,11]. Soil microbial number and microbial biomass, which serve as important indicators for assessing soil microbial activity, are equally regulated by soil environmental conditions. Carbon (C) and nitrogen (N) elements in soil are the cornerstones of microbial growth and metabolism, which are closely linked to microbial reproduction and metabolic activities. When soil C and N are more abundant, the MBC content is also higher [12]. Du et al. [13] also reached the same conclusion in their study of citrus orchard soils, indicating that microorganisms are able to absorb and utilize these nutrients more efficiently under conditions of sufficient C and N sources, which, in turn, promotes their growth and metabolic activity, which is ultimately reflected in the increase in microbial number. Soil pH is also widely recognized as a major environmental factor capable of directly or indirectly influencing changes in microbial carbon metabolism [14,15,16]. For example, soil pH can affect the relative abundance of bacteria: as soil pH increases, the relative abundance of actinobacteria increases, while that of acidobacteria and verrucose microorganisms decreases [17], which in turn affects carbon source metabolic activity and diversity of microcommunities. Specific elements such as Ca^2+^ also play an important role in soil microbial metabolic activities. Ca^2+^ acts as an activator for some enzymes that significantly improves cellular metabolism [18,19] and enhances the activity of some specific enzymes such as sucrose metabolizing enzymes [20]; therefore, higher Ca^2+^ content may favor the metabolism of carbon sources by microorganisms. The results of previous studies showed that Ca^2+^ had a significant positive effect on microbial communities and diversity in forests and orchard soils. Ca^2+^ plays a crucial role in biological processes, being able to influence the process of cell division, gene expression, and cell differentiation [21], and Ca^2+^ promotes microbial proliferation and activity by improving the cell cycle and metabolic pathways [19]. Increased soil potassium (ion K^+^) has been shown to significantly enhance microbial number and microbial biomass carbon (MBC) in agricultural soils [22]. Ion K^+^ is not only an important nutrient for plant growth, but also has a significant effect on soil microbial life activities, such as being involved in the regulation of protein synthesis, enzyme activation and other processes [23], so the appropriate range of increasing the ion K^+^ content may be able to promote microbial growth and metabolism [16,24]. These scattered studies have shown that pH, soil C, soil N, soil K^+^ and Ca^2+^ can regulate microbial vitality and carbon metabolism diversity. However, these studies mostly focus on the analysis of a single soil type or microbial community structure [25,26]. Systematic comparison of microbial carbon source metabolic diversity among different types of soils and its relationship with soil properties are still insufficient, especially in the specific ecosystem of citrus orchards, where the study on soil microbial carbon source metabolic diversity is rarely reported. Therefore, we chose three soil types where citrus is grown to investigate the differences in soil microbial carbon metabolism characteristics between karst and non-karst soils and their environmental impact factors.

Due to its special geological background and soil-forming characteristics, the soil of KR is more alkaline and rich in Ca^2+^ than that of the non-karst region (NKR) [27], and the SOC in KR is generally higher. Such special properties may have an impact on the carbon metabolism diversity of soil microorganisms, making them different from soil microorganisms in the NKR. Previous studies have revealed that KR soils harbor complex microbial communities that are different from those of NKRs [28,29], suggesting that the karst environment has an important influence on microbial community. Previous studies have confirmed that pH, Ca^2+^, SOC and TN are key factors affecting soil microbial diversity [30], microbial number [31] and microbial biomass [32] in KRs, indicating that the accumulation of SOC and TN and suitable pH levels and mineral element content jointly shape the specific soil microbial community structure and function in KR. These microbial communities are not only different in species and numbers from NKR, but also show their unique metabolic activities and ecological functions. It is worthwhile to continue studying which environmental factors make the microbial diversity of soils in KRs different from those in NKRs. Therefore, this study raises the following scientific questions: (1) Is the diversity of microbial carbon metabolism in KR soil significantly different from that in NKR soil? (2) What are the key environmental factors influencing the characteristics of soil microbial carbon metabolism? To address these two problems, this study took citrus orchard soil in the KR, NKR and MR (mixed region) of southern China as the research object to measure and compare the microbial number, microbial biomass, microbial carbon metabolism activity and diversity of different types of soil microorganisms. The aim of this study is to provide scientific data and theoretical support for understanding the uniqueness of soil microbial carbon metabolism in KR and the environmental drivers behind it, to enhance our understanding of microbial-driven carbon cycling processes, which holds both academic and practical value in the context of global climate change and carbon reduction goals. Furthermore, we propose the research hypothesis that pH and Ca^2+^ influence the carbon metabolic characteristics of microbial communities by enhancing and altering the “quality” and “quantity” of the soil.

## 2. Materials and Methods

### 2.1. Overview of the Study Area

The long-term experimental site of this study is located in the karst experimental field in Mao Village, Chaotian Township, Guilin City, Guangxi Zhuang Autonomous Region (110°31.756′–110°35.116′ E, 25°07.820′–25°11.482′ N) which belongs to the subtropical monsoon zone, with an average annual temperature of 18.8 °C and an average annual rainfall of 1915.2 mm (Figure S2). The region has a long geological history and a high intensity of karst landscape development, which is a typical representative of subtropical karst topography. The unique geological background and climatic conditions of this study area make it a unique natural laboratory for exploring the effects of soil physicochemical properties on the characteristics of microbial carbon metabolism and diversity.

The experimental site includes three main types of regions: KR, MR and NKR. The soil in the KR region is brown limestone soil formed by weathering of carbonate rocks. According to the USDA Soil Taxonomy, it falls under the category of inceptisols (aquepts). Soil in NKR consists of red soil derived from sandy shale, which may be classified as ultisols according to the USDA Soil Taxonomy. The natural vegetation primarily features oak-dominated forests. And some transitional soils exist between KR and NKR.

The typical citrus varieties sampled are *Citrus reticulata* ‘*Unshiu*’ and *Citrus reticulata* ‘*Kinokuni*’. The age of the citrus trees was around 6–8 years. Compound fertilizer was spread in March, May, July, and November every year at a dosage of 0.3–0.5 kg per tree.

### 2.2. Soil Sampling and Pre-Treatment

In December 2013, soil samples were collected from citrus orchards in the KR, MR and NKR in the karst experimental field of Guilin, Guangxi Zhuang Autonomous Region, respectively, and a total of three soil samples were collected from the KR, three from the MR and three from the NKR. The sampling area consists of a square with sides of 1 m, extending approximately 50 cm on either side of the vertical drip line of each tree’s canopy. Soil samples were collected from the surface soil layer of 0 to 20 cm depth, each quadrat covered an area of approximately 1 m^2^. The three adjacent aliquots were homogeneously mixed in the field using the method of coning and quartering to form one soil sample. In Guangxi, citrus planting occurs densely. The planting spacing is about 2.5 m. The canopy diameter ranges from 1.5 to 2 m. The sampling range for each plot extends 50 cm outside and inside the vertical drip line of the canopy. Thus, the soil includes portions under and between the canopies. The soil samples collected on the same day were returned to the laboratory, where extraneous materials such as plant roots and debris were removed. The samples were naturally air-dried in a dark place indoors (moisture content is 40%), one portion of the samples was ground through a 2 mm sieve and stored in a 4 °C refrigerator for measuring dissolved organic carbon (DOC), microbial biomass carbon (MBC) and nitrogen (MBN), microbial number, and microbial carbon source metabolism. The other portion continued to be air-dried and was subsequently ground through a 0.15 mm sieve for the analysis of soil physicochemical properties.

### 2.3. Soil Physicochemical Properties Analysis

Soil physicochemical properties were analyzed by routine methods [33,34].

The soil moisture content (W) was determined using the gravimetric method, with the calculation formula as follows:

(1) $$\begin{array}{c} W=(B-C)/(B-A) \end{array}$$

A—weight of the aluminum container after drying (g);

B—weight of the soil sample and aluminum container before drying (g);

C—weight of the soil sample and aluminum container after drying (g).

Soil pH was determined by pH meter (PHS-3E type, China).

Soil organic carbon (SOC) was determined by concentrated sulfuric acid–potassium dichromate external heating method [35].

Soluble organic carbon (DOC) was determined by soil and water shock extraction method using total organic carbon analyzer (multi N/C 3100, Analytik Jena, Germany).

Total nitrogen (TN) was determined by concentrated sulfuric acid digestion and Kjeldahl method [36].

C/N ratio was calculated as follows:

(2) $$\begin{array}{c} C/N=SOC∕TN \end{array}$$

Alkaline nitrogen (AN) was determined by the NaOH-hydrolyzing, NH_3_-diffusing, H_3_BO_3_-absorption method [37].

Available phosphorus (AP) was extracted using sodium bicarbonate and determined by UV Spectrophotometer (UV-9000, METASH, China).

Available potassium (AK) was extracted using ammonium acetate and determined by Atomic Absorption Spectroscopy (PinAAcle 900T, PerkinElmer, Commonwealth of Massachusetts, USA).

Cation exchange capacity (CEC) was determined by the ammonium acetate method [38].

Exchangeable calcium (Ca^2^⁺) and exchangeable magnesium (Mg^2^⁺) were extracted using ammonium acetate and determined by Inductively Coupled Plasma Optical Emission Spectrometer (Optima 7000 DV, PerkinElmer, Commonwealth of Massachusetts, USA).

### 2.4. Determination of Soil Microbial Biomass Indicators

#### 2.4.1. Soil Microbial Number

Fresh soil samples of 10.0 g were weighed and put into a triangular flask together with 90 mL of sterile water and shaken in a reciprocating shaker for about 20 min to mix thoroughly to obtain 10^−1^ soil bacterial suspension. Then, this was sequentially diluted into different dilutions (10^−2^, 10^−3^, 10^−4^, 10^−5^) of the soil solution, and then inoculated with a sterile pipette snatch to suck up 200 μL of different dilutions of the soil bacterial solution; each dilution gave three replicates of samples, all using the plate coating method, which were placed in a constant temperature incubator culture. Soil microbial number was detected by the plate coating method. Bacteria were cultured with beef extract peptone AGAR medium [39] and cultured at 37 °C for 2 days (dark conditions, the same below). Actinobacteria were cultured with Gao’s No. 1 medium [40] and cultured at 25 °C for 3 days; fungi were cultured with Martin’s medium [41] and cultured at 28 °C for 2 days. The dilution with a colony count between 30 and 300 was used as the standard for counting bacteria and actinobacteria, and the dilution with a colony count between 10 and 100 was used as a standard for fungi. Calculation results are expressed as the number of colonies per gram of dry soil.

Calculation formula:

(3) $$\begin{array}{c} Number\;of\;colonies\;per\;gram\;of\;dry\;soil=Average\;number\;of\;colonies\times Dilution\;factor\times (1-W) \end{array}$$

#### 2.4.2. Soil Microbial Biomass Carbon and Nitrogen

Soil microbial biomass was determined by the chloroform fumigation method [42].

Calculation formulas:

Soil microbial biomass carbon (MBC):

(4) $$\begin{array}{c} MBC=EC∕k_{EC} \end{array}$$

*EC*—Difference between organic carbon of fumigated and unfumigated soil samples, mg/kg.

*k_EC_*—The proportion of C leached out of microorganisms killed by chloroform fumigation is generally taken as 0.38 [43].

Soil microbial biomass nitrogen (MBN):

(5) $$\begin{array}{c} MBN=EN/k_{EN} \end{array}$$

*EN*—Difference between total nitrogen of fumigated and unfumigated soil samples, mg/kg.

*k_EN_*—The proportion of N in microorganisms killed by chloroform fumigation that is extracted by K_2_SO_4_ is generally taken as 0.54 [43].

Microbial carbon-to-nitrogen ratio: MBC/MBN.

#### 2.4.3. Soil Microbial Quotient

Calculation formula:

Soil microbial quotient (SMQ):

(6) $$\begin{array}{c} SMQ=MBC/SOC \end{array}$$

### 2.5. Soil Microbial Utilization of Carbon Sources

The following process was carried out. Weigh 10 g of fresh soil sample and place it in a triangular flask with 90 mL of sterile water. Agitate the mixture in a shaker for 30 min at 120 revolutions per minute (r·min^−1^). Subsequently, on a laminar flow hood, use a 10-fold dilution method with sterile water to dilute the mixture to 10^−3^ (ensure thorough agitation after each dilution). After a 20 min ice-water bath, pipette the upper layer of the soil suspension into ECO microplates, with 150 μL per well. Place the inoculated ECO microplates in a constant temperature incubator at 28 °C for continuous cultivation for 240 h. Collect the absorbance values at 590 nm and 750 nm every 12 h [44].

The BIOLOG ECO microplate was used to analyze the microbial utilization of carbon sources. The ECO plate consists of 96 small wells, where every 32 wells are a replicate, containing 31 kinds of carbon sources and one blank control. These wells are filled with various carbon sources and dyes. As microorganisms utilize the carbon sources, their reaction with the dye causes color changes in the wells. The alteration of color can be utilized as an indicator of the extent to which microorganisms utilize the carbon sources [45].

### 2.6. Data Analysis

#### 2.6.1. Statistical Analysis

We calculated and organized the data in EXCEL.

Descriptive statistical analysis, one-way ANOVA, LSD test (minimum standard deviation test, *p* = 0.05) and correlation analysis were carried out on the measured data with SPSS 26.0 statistical software.

Rstudio’s (R 4.3.3) corrplot and pheatmap packages were used to perform correlation analysis and significance testing and draw heatmaps.

#### 2.6.2. The Calculation Formula of Soil Microbial Carbon Source Metabolism and Community Functional Diversity

Calculate the Average Well Color Development (AWCD), Shannon Index (*H*′), Shannon-evenness Index (*E*), Simpson Index (*D*) and McIntosh Index (*U*) based on the referenced literature [46,47].

Calculation formula:

(7) $$\begin{array}{c} AWCD=\sum \left(C_{i}-R\right)/31 \end{array}$$

(8) $$\begin{array}{c} P_{i}=(C_{i}-R)/\sum \left(C_{i}-R\right) \end{array}$$

(9) $$\begin{array}{c} H^{\prime }=-\sum P_{i}\times \ln P_{i} \end{array}$$

(10) $$\begin{array}{c} E=H^{\prime }/\ln S \end{array}$$

(11) $$\begin{array}{c} D=1-\sum P_{i}^{2} \end{array}$$

(12) $$\begin{array}{c} U=\sqrt{{\sum \left(C_{i}-R\right)}^{2}} \end{array}$$

*C_i_* is the optical density difference in each reaction well at 590 nm and 750 nm; *R* is the average of optical density of three replicates with the same substrate on the ECO plate at wavelengths of 750 nanometers and 590 nanometers; *C_i_* − *R* ≥ 0 (if *C_i_* − *R* < 0, it is calculated as 0). *P_i_* is the absorption as a fraction of the total absorption of all wells. *S* is the number of wells showing color development.

## 3. Results

### 3.1. Comparison of Physicochemical Properties and Nutrients of Soils in Karst and Non-Karst Regions

The data in Table 1 show that SOC, pH, and exchangeable Ca^2+^ in the KR soil were significantly higher than those in the other two soils. However, there were no significant differences in DOC, TN, AN, C/N, AP, AK, CEC and exchangeable Mg^2+^ among the three regional soils.

### 3.2. Comparison of Soil Microbial Number and Biomass Between Karst and Non-Karst Regions

#### 3.2.1. Soil Microbial Number

According to the data in Table 2, it can be observed that the number of bacteria in soil of KR is significantly higher than that in the MR and NKR, while there is no significant difference in the number of fungi and actinobacteria among the three regions.

#### 3.2.2. Soil Microbial Biomass

Soil microbial biomass is an important indicator of microbial metabolism, and SMQ represents the amount of MBC per unit of organic carbon. According to the data in Table 3, the MBC and SMQ in KR were significantly higher than those in NKR, while there were no significant differences in MBN and MBC/MBN among the three different regional soils.

### 3.3. Soil Microbial Carbon Source Metabolic Diversity

The BIOLOG ECO microplate has three replicates, each with 31 carbon sources as shown in Table S1. The 31 carbon sources are categorized into six major groups: Carbohydrate, Polyols, Amines, Esters, Amino acids, and Carboxylic acids. The positional distribution of each type of carbon source on the ECO plate is shown in Figure S1.

#### 3.3.1. Soil Microbial Carbon Source Metabolic Activity

**Figure 1:**
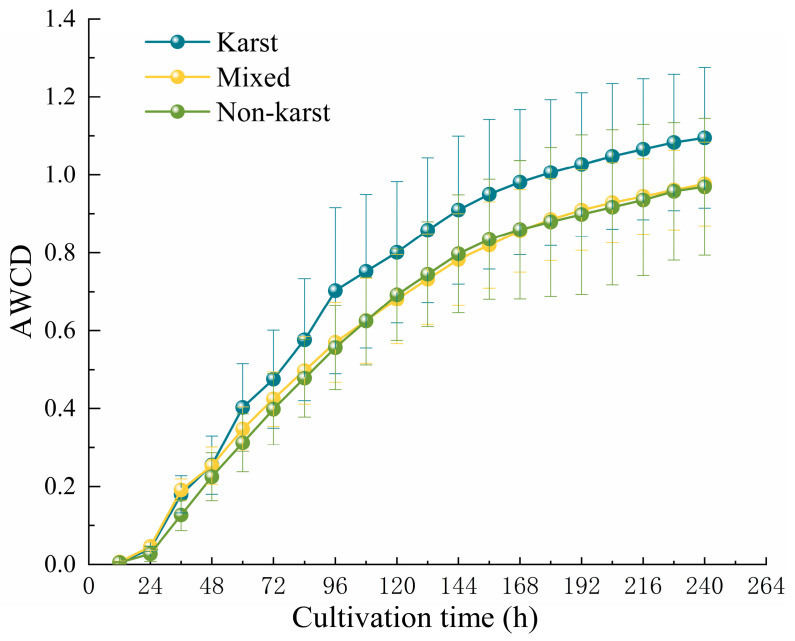
Average Well Color Development (AWCD) of Soil microbial communities in KR, MR, and NKR of citrus orchards.

The AWCD is used to represent the overall activity of microbial carbon source metabolism. As shown in Figure 1, the AWCD values of the three different regional soils exhibit a continuous growth trend with the extension of cultivation time, indicating that the metabolic activity of microorganisms increases with the increase in cultivation time. In the initial cultivation phase (0–24 h), the AWCD growth rate is relatively slow. This indicates that the utilization efficiency of microorganisms for carbon sources is low during this stage, and they are in the adaptation phase to the growth environment, with their metabolic activity not fully activated. At 24 h, the AWCD change rate shows a significant inflection point. In the subsequent early cultivation phase (24–144 h), the AWCD change rate continues to increase. This indicates that microorganisms begin to utilize carbon sources on a large scale to support their life. The microorganisms have successfully adapted to their growth environment and entered the logarithmic growth phase, with their metabolic efficiency and growth rate significantly increased. In the late cultivation phase (144–240 h), the AWCD change rate gradually decreases, and in the final phase (after 240 h), it tends to stabilize. This indicates that the growth rate of microorganisms slows down, and the activity of carbon source metabolism reaches a relatively balanced state, with microorganisms entering the stable growth phase. Compared with the three regional soils, the AWCD values and growth rates of the KR are higher than those of the MR and NKR. After 240 h, the AWCD value of the KR reached 1.095, while the AWCD values of the MR and NKR are only 0.9763 and 0.969, respectively. This indicates that the carbon metabolic ability and activity of soil microorganisms in the KR are higher than those in other two soils.

#### 3.3.2. Soil Microbial Community Functional Diversity

**Figure 2:**
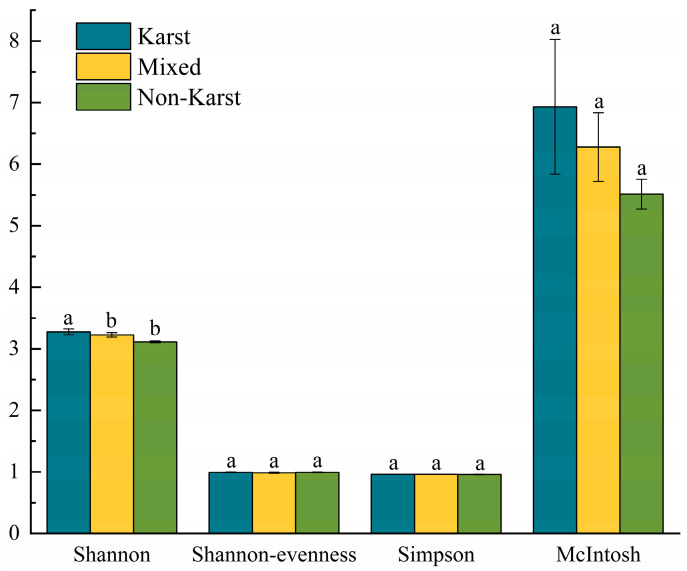
Microbial Community Functional Diversity Index. Different letters denote significant differences; *p*< 0.05; a > b.

The data of soil microbial cultivation at 240 h were selected and analyzed to characterize the four diversity indices of soil microorganisms in the three regions. *H*′ primarily measures the diversity of microbial communities. *E* is specifically used to measure the evenness of communities. *D* primarily measures the concentration or dominance of species in communities. *U* aims to measure the diversity of communities, particularly the evenness of species distribution. The results are shown in Figure 2 and Table S2. The *H*′ of soil microorganisms in the KR was significantly higher than that in the NKR. This suggests that soil microorganisms in the KR have richer diversity and more complex metabolic functions.

#### 3.3.3. Soil Microbial Carbon Source Utilization Intensity

**Figure 3:**
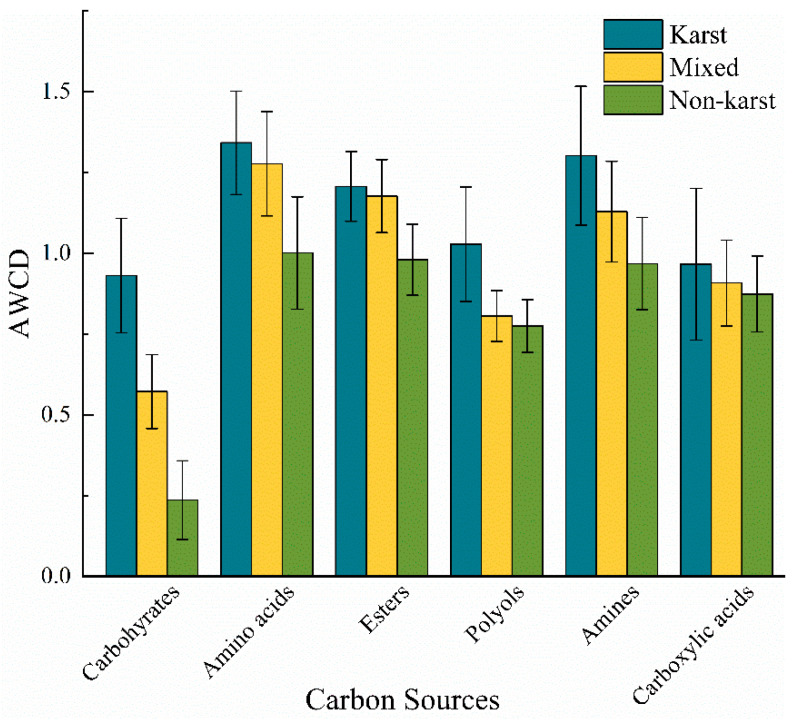
Utilization of six major groups of carbon sources by soil microorganisms in KR, MR, and NKR of citrus orchards.

The BIOLOG ECO microplate contains 31 carbon sources, categorized into six groups: Carbohydrate, Polyols, Amines, Esters, Amino acids, and Carboxylic acids. As shown in Figure 3, the AWCD values of various types of carbon sources all exhibit a trend of KR > MR > NKR. The number of carbon sources with AWCD > 1.0 is KR (4) > MR (3) > NKR (1) (Table S3 and Figure 3), indicating that microorganisms in the KR have a higher utilization rate of the six categories of carbon sources than the other two. The top three carbon sources utilized by microorganisms in the KR are Amino acids > Amines > Esters; those in the MR and NKR are: Amino acids > Esters > Amines, suggesting that the dominant microbial communities in all three regional soils are all composed of Amino acids, Amines, and Esters.

#### 3.3.4. Relationship Between Soil Microbial Number, Community Diversity Index, and Metabolic Activity with Soil Physicochemical Properties

The analysis explored the correlations between the soil microbial number, metabolic activity, diversity indices, and physicochemical properties of the three regional soils. The correlation heatmap showed that pH was significantly positively correlated with bacterial number, actinobacteria number, and *H*′ (*p* < 0.05). SOC was extremely significantly positively correlated with bacterial number and *U* (*p* < 0.01), and significantly positively correlated with actinobacteria number, MBC, *H*′, and AWCD (*p* < 0.05). TN was significantly positively correlated with MBC and *E* (*p* < 0.05). AN was significantly positively correlated with *E* (*p* < 0.05). AK was significantly positively correlated with bacterial number and MBC (*p* < 0.05). Exchangeable Ca^2+^ was extremely significantly positively correlated with actinobacteria number (*p* < 0.01), and significantly positively correlated with bacterial number, MBC, and *H*′ (*p* < 0.05). Exchangeable Mg^2+^ was significantly negatively correlated with fungal number (*p* < 0.05). The above research results indicate that pH, SOC, TN, exchangeable Ca^2+^, and AN and AK are the main influencing factors causing differences in carbon metabolic characteristics of microorganisms among the three regional soils. Among them, pH, SOC, TN, AK, and exchangeable Ca^2+^ are the key environmental factors that differentiate the soil microbial carbon metabolism profiles among the three regions.

## 4. Discussion

### 4.1. Influence Factors of Soil Microbial Quantity and Biomass in Karst Regions

Soil microorganisms play a crucial role in driving the carbon cycle and are significant contributors to carbon balance [1]. An increased abundance of these microorganisms enhances their decomposition abilities, thereby facilitating the conversion and cycling of carbon. In this study, the number of bacteria and actinobacteria in the KR was significantly higher than that in the NKR and MR (*p* < 0.05) (Table 2), and there was a significant positive correlation between pH and microbial number. This indicates that the higher soil pH in the KR had a certain positive impact on microorganisms. In citrus orchards, soil acidification is an unavoidable trend due to long-term fertilization, and monoculture can lead to the accumulation of organic acids, causing soil acidification [48]. In acidic soils, the solubility of toxic metal ions such as Al^3+^, Cu^2+^, and Cd^2+^ increases, which is detrimental to microbial survival [49]. However, the soil parent material characteristics of KR are just able to neutralize this soil acidification trend and mitigate its harm. Furthermore, the increase in pH promotes the solubility of nutrient elements in the soil and their availability to the plant (such as phosphorus, calcium, and magnesium) [50,51], improving the microbial survival environment and thereby promoting microbial growth.

MBC is a key indicator of soil microbial activity [8,52] and its content in KR soil was significantly higher than that in NKR (*p* < 0.05) (Table 3), suggesting that SOC is the main source of energy and matter for microbial growth and metabolism. MBC/MBN did not show significant differences among the three soil types, possibly because lithology is not a key indicator affecting MBC/MBN [53,54], and the C/N ratio of microbial biomass is generally a relatively stable value. SMQ can reflect the efficiency of SOC conversion to MBC, and a faster SOC decomposition rate indicates a faster conversion of active organic carbon, which is accompanied by more active soil microorganisms. SMQ is an important indicator of soil quality. The SMQ of KR soil was significantly higher than that of NKR soil (*p* < 0.05) (Table 3). On one hand, at the community level, the combination of Ca^2+^ with humic acids in soil forms stable calcium humate [55]. This not only elevates soil pH and alters the microbial niche but also decreases the availability of organic matter to microorganisms, thereby driving changes in soil microbial community structure and functional evolution. Some microorganisms may continue to adapt to high-calcium environments, while others, under limited resource conditions (with no significant difference in DOC among the three regions), must maintain high utilization efficiency and turnover rates. On the other hand, at the individual level, Ca^2+^ may enhance the carbon utilization efficiency of certain microorganisms by influencing their carbon assimilation pathways, electron transport systems, and the tricarboxylic acid (TCA) cycle [56]. The higher SMQ also indicates a faster turnover rate of SOC in KR. It further confirms that microorganisms in KR soil not only have a large population, but also have stronger carbon source degradation and transformation ability. SOC is a crucial environmental parameter that influences the carbon metabolism of microbial communities [57]. In this study, SOC shows a significant positive correlation with both soil microbial number and MBC. SOC is the main energy and carbon source for microorganisms [7]. Microorganisms obtain energy by decomposing organic carbon, which enables them to grow and reproduce. Therefore, an increase in SOC content directly provides more energy and carbon sources for microorganisms, promoting their growth. Moreover, different microbial species have different strategies for utilizing organic carbon, and under high SOC levels, the microbial community structure may become more diverse and complex. This complex community structure can more effectively promote carbon cycling and storage. Moreover, microbial metabolic activities generate new organic compounds [58], which can be reused by other microorganisms or plant roots, forming a well-established carbon cycling network, thereby promoting microbial reproduction and the accumulation of microbial biomass.

N is also an essential nutrient element that affects microbial life activities. In this study, N shows a significant positive correlation with MBC. Firstly, N is a basic element for microorganisms to synthesize proteins, nucleic acids, and other biomacromolecules [59], which is crucial for microbial growth and metabolism. An adequate N source can promote microbial growth and metabolic activities, directly influencing microbial utilization and transformation of carbon sources, and ultimately affecting the accumulation of MBC. As many enzymes are proteins themselves, an increase in N can stimulate the activity of some carbon metabolic enzymes, such as β-1,4-glucosidase [60], thereby accelerating the microbial metabolic processes, ultimately resulting in an increase in MBC.

In this study, AK showed a significant positive correlation with bacterial number and MBC. Firstly, AK is essential for plant growth, particularly for promoting root growth [61]. AK may indirectly improve the microbial survival environment by promoting plant root growth, thereby improving microbial biochemical activity. Secondly, microorganisms can convert K that is difficult to be absorbed by plants into AK that is easily absorbed [62], which can explain the mutual promotional relationship between microbial metabolic activity and soil AK content. Additionally, K is an activator of many enzymes within microorganisms, which can promote enzyme protein synthesis [63,64], further increasing microbial carbon utilization efficiency.

The calcium-rich characteristic of KR is also a crucial factor influencing microbial number and MBC. In this study, exchangeable Ca^2+^ was significantly positively correlated with microbial number and MBC. Firstly, Ca^2+^ can form stable humus–calcium complexes with organic matter (humus) through ionic bonds, providing microorganisms with a slow-release but stable carbon source, which is beneficial for microbial reproduction, metabolism, and SOC accumulation [65]. The research results of Babur et al. [54] also showed that the microbial community in karst soil exhibited higher bioactivity and metabolic efficiency, which further supports the results of this study. Secondly, Ca^2+^ is an essential macronutrient for plants. Morales et al. [66] found that applying calcium fertilizer promoted the growth of citrus roots. Therefore, the calcium-rich soil environment in KR may be beneficial for citrus. Additionally, the litter and root exudates of citrus are also carbon sources for microorganism [67]. This contributes to the higher microbial number and more active carbon metabolic activities in KR.

### 4.2. Influence Factors of Carbon Source Metabolism Diversity of Soil Microorganisms in Karst Regions

Soil environment can affect the diversity of microbial community structure and function [54,68]. In this study, the SOC, pH, and exchangeable Ca^2+^ in KR soil were significantly higher than those in MR and NKR soils (Table 1). The correlation analysis results also showed that SOC, pH, and exchangeable Ca^2+^ were the main factors influencing the differentiation of microbial carbon metabolism profiles among the three regional soils, indicating that the karst environment promotes the carbon metabolic function and diversity of microorganisms.

**Figure 4:**
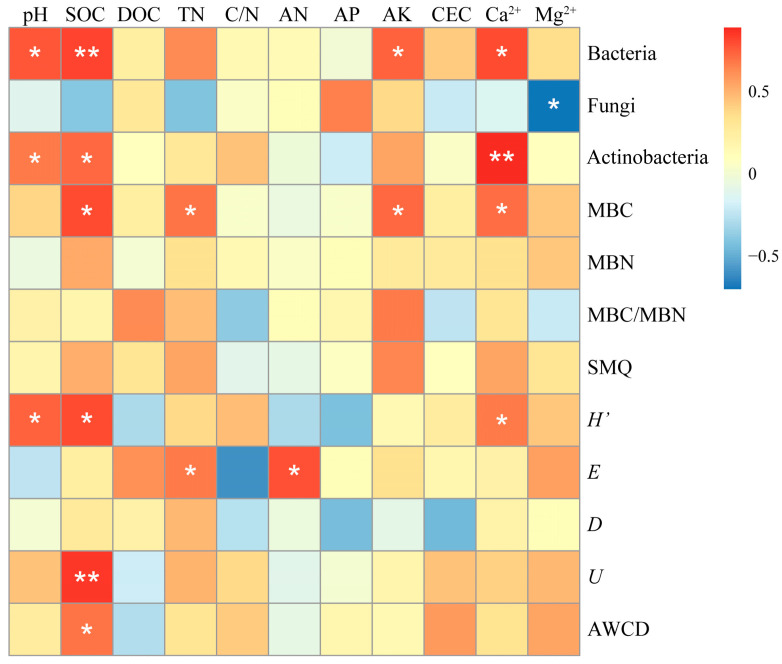
Heatmap of correlations between soil microbial diversity indices, metabolic activity, and soil physicochemical properties. Note: * *p* < 0.05 (Pearson correlation, two-tailed), ** *p* < 0.01 (Pearson correlation, two-tailed).

The Shannon Index (*H*′) is correlated with the number of carbon substrates that can be degraded by microbial communities [69]. The Shannon-evenness Index (*E*) and McIntosh Index (*U*) are concerned about the evenness of all the utilized substrates [70], while the Simpson Index (*D*) primarily reflects the dominance of species [44]. By combining these four diversity indices, the carbon metabolic diversity and characteristics of microorganisms can be assessed. In this study, the *H*′ of soil microorganisms in the KR was significantly higher than that in the NKR (*p* < 0.05) (Table S2, Figure 3). Considering the AWCD values, the types of carbon source metabolism in KR are more abundant, and the carbon source utilization rate is higher, implying ecological significance such as promoting nutrient cycling [71], enhancing ecosystem stability [72], and improving soil functionality [73]. Of course, a more detailed and precise analysis can be conducted by combining high-throughput sequencing, metabolomics, and other technologies. pH, SOC, TN, AN, and exchangeable Ca^2+^ were the main environmental factors that contributed to the differences in microbial community diversity and evenness between KR and NKR soils (Figure 4). The research results of Liu et al. [16] and Hamidovic et al. [74] showed that pH and carbon content have a significant impact on the quantity and structure of soil microorganisms. In nutrient-rich environments, microbial metabolism is vigorous, and reproduction is rapid [75,76]. Interspecific competition for resources is relatively weak, which is more conducive to the co-prosperity of microorganisms. pH has a selective effect on microbial assembly. For example, actinobacteria can reproduce faster in neutral to alkaline soils [17,77]. The higher SOC content and pH in KR may be more suitable for microbial growth and reproduction. N can affect microbial community, which is confirmed by the positive correlation between soil TN and AN content and the *E*. The research results of Luo et al. [10] also support the conclusions of this study, as the microorganisms’ diversity increases with the increase in N in the soil. The possible reason is that nitrogen can promote plant growth and increase the amount of plant litter and underground biomass [78]. The large amount of organic matter returned to the soil may be the reason for the increased functional diversity of microbial communities. Ca^2+^ is an important second messenger in biological cells, and when combined with calmodulin (CaM), it regulates the activity of many key enzymes and metabolic processes in cells. It also plays an important role in the soil microorganisms’ metabolic processes. For example, Li et al. [79] found that Ca^2+^ increased the activity of ATPase and amine oxidase, indicating that the higher Ca^2+^ levels in karst regions can promote microbial life metabolism by enhancing the activity of certain enzymes, thereby improving microbial carbon metabolic abilities.

As evaluated by AWCD values, it was found that the utilization rates of the six major carbon sources by soil microorganisms in the KR were higher than those in the MR and NKR during the 240 h cultivation period (Figure 3), exhibiting stronger microbial carbolic metabolism. This is closely related to the richer nutrients, higher microbial number, and greater microbial diversity in the KR. Amino acids, Amides, and Esters are the three carbon sources with the highest utilization efficiency in the three regional soils studied in this research, among which amino acids have the highest utilization efficiency (Figure 3). There are two possible reasons for this. Firstly, Amino acids are low-molecular-weight organic compounds [14] with relatively simple molecular structures, which reduces the chemical barrier faced by microorganisms during the utilization processes. Therefore, microorganisms can obtain the required carbon and energy sources from Amino acids more efficiently. Secondly, Amino acids are the main components of citrus tree root exudates [80]. As an arbor plant, citrus has a well-developed root system that can secrete large amounts of Amino acids into the soil. This not only provides a rich carbon source for microorganisms but also promotes their adaptation and preference for Amino acid-based carbon sources, making Amino acids the most utilized carbon source in microorganisms.

The differences in carbon source utilization between soil microorganisms in KR and NKR are manifested in the utilization efficiency of Amine and Ester carbon sources. In KR soil, microorganisms have a higher utilization efficiency of Amine carbon sources compared to Ester carbon sources. This discrepancy may be attributed to the decarboxylation process, an intermediate step in microbial metabolism of Amino acids, which produces Amines [81]. The higher utilization efficiency of Amino acids by soil microorganisms in KR promotes the accumulation of Amine substances within the soil, thereby enhancing the availability of Amine carbon sources. During the adaptation process of microorganisms to their environment, they may have developed a preference and high-efficiency utilization ability for Amine carbon sources. This adaptability renders microorganisms in KRs more inclined to utilize Amines when presented with a variety of carbon source options. Additionally, previous studies have shown that Ca^2+^ can increase the activity of enzymes such as diamine oxidase (DAO) and polyamine oxidase (PAO) [79], indicating that the higher Ca^2+^ content in KR soil may enhance the activity of Amine oxidases, leading to a higher utilization efficiency of Amine carbon sources. Future research should consider applying N stable isotope tracing technology to further clarify the intrinsic mechanisms of rapid utilization of Amines by soil microorganisms in karst regions [82].

## 5. Conclusions

This study investigates the differences in microbial carbon metabolism capacity and diversity between karst and non-karst soils. Through comparative analysis, we found that karst soils significantly outperform non-karst soils in terms of microbial carbon metabolism capacity and diversity. Additionally, the number of bacteria, microbial biomass carbon (MBC), and soil microbial quotient (SMQ) in karst soils were all significantly higher than those in non-karst soils. Our findings indicate that pH, soil organic carbon (SOC), and exchangeable Ca^2+^ are the primary factors contributing to the differences in microbial carbon metabolism characteristics between karst and non-karst soils, with Ca^2+^ playing a crucial role in regulating the interaction between soil microbial diversity and functionality with carbon sequestration. This further suggests that adding an appropriate amount of Ca^2+^ to soils with lower pH is an effective method for increasing soil carbon accumulation. Furthermore, Ca^2+^ contribute to the reduction of carbon dioxide (CO_2_) emissions from soil to the atmosphere by enhancing the fixation of soil carbon, thereby mitigating climate change. This has significant implications for agricultural production and ecological restoration practices, as it offers a potential soil management strategy that can enhance the carbon sink function of soil and promote plant growth. In the context of global climate change and the pursuit of sustainable development goals, this strategy is particularly crucial. Future research could further employ stable isotope tracing methods to explore the effects of Ca^2+^ on soil microbial diversity and carbon metabolism under different soil types and environmental conditions, as well as how these effects may influence ecosystem services and agricultural production in the long term.

## Figures and Tables

**Table 1:** Physicochemical properties in KR, MR and NKR.

Regions	pH (H_2_O)	SOC (g/kg)	DOC (mg/kg)	TN (g/kg)	C/N	AN (mg/kg)	AP (mg/kg)	AK (mg/kg)	CEC (cmol/kg)	Exchangable Ca^2+^ (cmol/kg)	Exchangable Mg^2+^ (cmol/kg)
KR	7.60 ± 0.30 a	17.69 ± 2.36 a	546.44 ± 24.20 a	0.77 ± 0.08 a	22.85 ± 2.07 a	62.99 ± 13.63 a	14.35 ± 6.60 a	1021.62 ± 116.34 a	11.08 ± 2.13 a	48.13 ± 22.80 a	5.04 ± 1.58 a
MR	6.90 ± 0.19 ab	13.81 ± 0.48 b	476.7 ± 59.49 a	0.60 ± 0.10 a	23.40 ± 4.16 a	60.79 ± 21.41 a	11.53 ± 4.39 a	506.79 ± 39.85 b	10.63 ± 0.54 a	7.58 ± 2.16 b	4.17 ± 1.51 a
NK	6.18 ± 0.69 b	13.11 ± 0.07 b	571.4 ± 77.46 a	0.64 ± 0.10 a	20.76 ± 3.29 a	74.95 ± 8.54 a	22.13 ± 4.95 a	833.56 ± 180.08 a	10.19 ± 2.00 a	4.08 ± 3.19 b	3.38 ± 1.35 a

Note: The data in the table are presented as mean ± SD. Different letters denote significant differences; *p* < 0.05; a > ab > b.

**Table 2:** Soil microbial number in KR, MR, and NKR.

Regions	Bacteria/×10^6^ CFU/g	Fungi/×10^3^ CFU/g	Actinobacteria/×10^5^ CFU/g
KR	5.69 ± 0.39 a	5.42 ± 1.56 a	3.02 ± 1.04 a
MR	4.41 ± 0.37 b	4.82 ± 2.18 a	2.19 ± 0.31 a
NKR	4.25 ± 0.71 b	8.41 ± 2.36 a	1.85 ± 0.19 a

Note: The data in the table are presented as mean ± SD. Different letters denote significant differences; *p* < 0.05; a > b.

**Table 3:** Soil microbial biomass, MBC/MBN, and microbial quotient in KR, MR, and NKR.

Regions	MBC (mg/kg)	MBN (mg/kg)	MBC/MBN	SMQ (%)
KR	608.24 ± 63.80 a	136.1 ± 50.63 a	4.74 ± 1.13 a	3.45 ± 0.18 a
MR	213.04 ± 80.01 b	73.89 ± 20.40 a	2.86 ± 0.45 a	2.75 ± 0.62 b
NKR	359.57 ± 79.75 b	117.21 ± 70.82 a	3.93 ± 2.29 a	2.58 ± 0.94 b

Note: The data in the table are presented as mean ± SD. Different letters denote significant differences; *p* < 0.05; a > b.

## Data Availability

The original contributions presented in this study are included in the article/Supplementary Material. Further inquiries can be directed to the corresponding author.

## Supplementary Materials

The following supporting information can be downloaded at: https://www.mdpi.com/article/10.3390/microorganisms13020267/s1, Table S1. 31 Carbon Sources on the BIOLOG ECO Plate. Table S2. Functional Diversity of Soil Microbial Communities in Karst, Mixed and Non-Karst Regions. Table S3. Utilization of Six Major Carbon Sources by Soil Microorganisms in Karst, Mixed, and Non-Karst Regions of Citrus Orchards. Figure S1. Schematic Distribution of the 6 Major Categories of Carbon Sources on the ECO Plate. Figure S2. Schematic Distribution of sampling locations.
