# Supplementary material for: Characterization of Microbial Carbon Metabolism in Karst Soils from Citrus Orchards and Analysis of Its Environmental Drivers
Source: Microorganisms. 2025 Jan 25;13(2):267. doi: 10.3390/microorganisms13020267 (PMC11857600; doi:10.3390/microorganisms13020267)
Supplement: Supplementary file 1 [file microorganisms-13-00267-s001.zip › microorganisms-3419924-supplementary.pdf]

Table S1. 31 Carbon Sources on the BIOLOG ECO Plate.

Carbon Sources	Position	Name	Carbon Sources	Position	Name
Esters	B1	Methyl pyruvate	Amino acids	A4	L(+)-Arginin
	C1	polysorbate 40		B4	L-Asparagine
	D1	Tween 80		C4	L-Asparagine
	A3	D-(-)-galactonic acid-γ-lactone		D4	L-Serine
Carbohydrate	E1	α-Cyclodextrin		E4	L-Threonine
	F1	glycogen		F4	GLYCYL-L-GLUTAMIC ACID
	G1	D-(+)-Cellobiose	Carboxylic acids	F2	D-Glucosamic acid
	H1	α-D-Lactose		B3	ALPHA-D-GALACTURONIC ACID HYDRATE
	A2	β-Methyl-D-Glucoside		C3	Salicylic acid
	B2	D(+)-Xylose		D3	4-Hydroxybenzoic acid
	G2	Glucose-1-phosphate		E3	4-Hydroxybutanoic acid
Alcohols	C2	I-Erythritol		F3	Itaconic acid
	D2	D-Mannitol		G3	α-Ketobutyric acid
	H2	D,L-α-Glycerol		H3	D(+)-Malic acid
Amines	E2	N-Acetyl-D-Glucosamine	CK	A1	No carbon source
	G4	Phenylethylamine			
	H4	1,4-butanediamine			

Table S2. Functional Diversity of Soil Microbial Communities in Karst, Mixed and Non-Karst Regions.

Area types	Shannon Index *H'*	Shannon-evenness Index *E*	Simpson Index *D*	McIntosh Index *U*
Karst	3.28±0.05a	0.99±0.01a	0.96±0.00a	6.93±1.10a
Mixed	3.23±0.04b	0.99±0.01a	0.96±0.00a	6.28±0.56a
Non-Karst	3.11±0.02b	0.99±0.01a	0.96±0.01a	5.51±0.24a

Note: The data in the table is presented as mean ± SD. Different letters denote significant differences; $p < 0.05$; a > b.

Table S3. Utilization of Six Major Carbon Sources by Soil Microorganisms in Karst, Mixed, and Non-Karst Regions of Citrus Orchards.

Regions	Carbonhyrates	Amino acids	Esters	Alcohols	Amines	Carboxylic acids
KR	0.93±0.18	1.34±0.16	1.21±0.11	1.03±0.18	1.30±0.22	0.97±0.24
MR	0.57±0.11	1.28±0.16	1.18±0.11	0.81±0.08	1.13±0.16	0.91±0.13
NKR	0.24±0.12	1.00±0.17	0.98±0.11	0.78±0.08	0.97±0.14	0.87±0.12

Note: The data in the table is presented as mean ± SD.

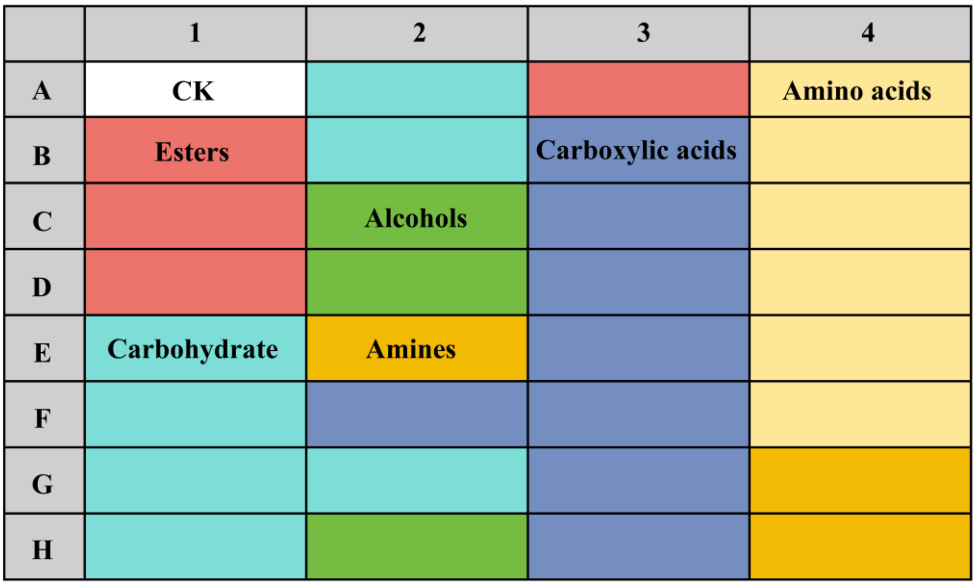

Figure S1. Schematic Distribution of the 6 Major Categories of Carbon Sources on the ECO Plate.

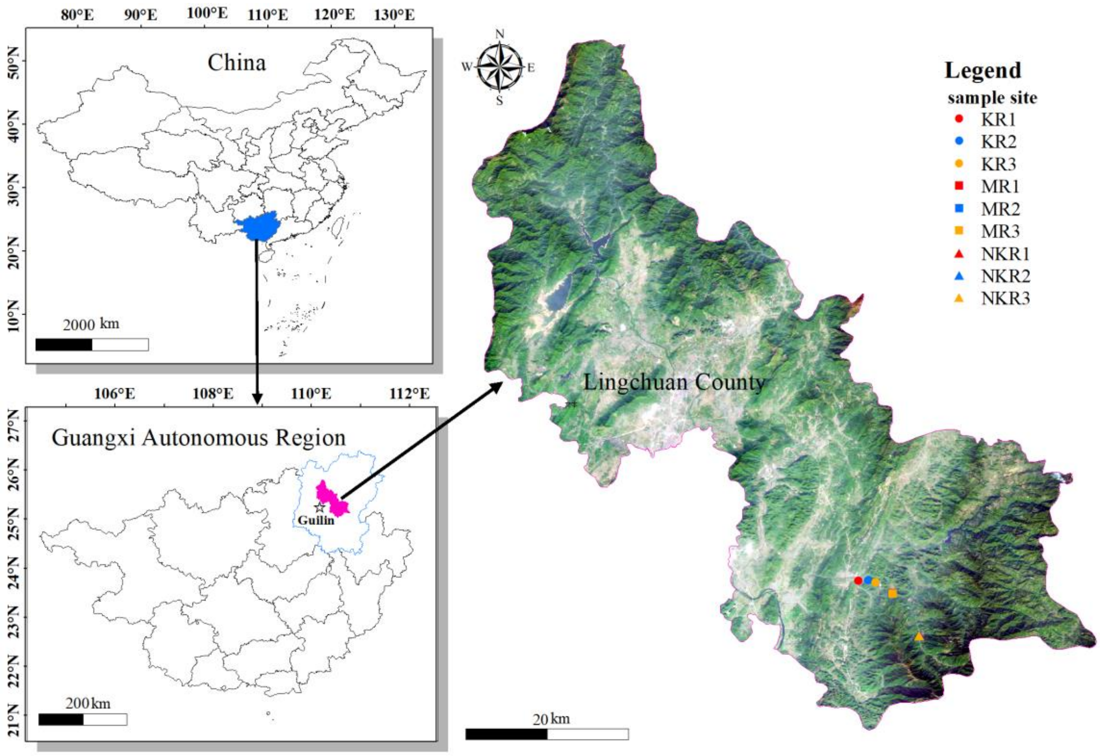

Figure S2. Schematic Distribution of sampling locations.
